# A Case of Adrenocorticotropin-dependent Cushing Syndrome with Osilodrostat Exposure in Early Pregnancy

**DOI:** 10.1210/jcemcr/luaf269

**Published:** 2025-11-19

**Authors:** Yukako Nakayama, Mayumi Ishikawa, Asako Watanabe, Yoshio Shima, Shigeyuki Tahara, Masato Iwabu

**Affiliations:** Department of Endocrinology, Diabetes and Arteriosclerosis, Nippon Medical School Musashi Kosugi Hospital, Kawasaki-shi, Kanagawa 211-8533, Japan; Department of Endocrinology, Metabolism and Nephrology, Graduate School of Medicine, Nippon Medical School, Bunkyo-ku, Tokyo 113-8603, Japan; Department of Endocrinology, Diabetes and Arteriosclerosis, Nippon Medical School Musashi Kosugi Hospital, Kawasaki-shi, Kanagawa 211-8533, Japan; Department of Endocrinology, Metabolism and Nephrology, Graduate School of Medicine, Nippon Medical School, Bunkyo-ku, Tokyo 113-8603, Japan; Department of Obstetrics and Gynecology, Nippon Medical School Musashi Kosugi Hospital, Kawasaki-shi, Kanagawa 211-8533, Japan; Department of Neonatal Medicine, Nippon Medical School Musashi Kosugi Hospital, Kawasaki-shi, Kanagawa 211-8533, Japan; Department of Neurological Surgery, Nippon Medical School Musashi Kosugi Hospital, Kawasaki-shi, Kanagawa 211-8533, Japan; Department of Endocrinology, Metabolism and Nephrology, Graduate School of Medicine, Nippon Medical School, Bunkyo-ku, Tokyo 113-8603, Japan

**Keywords:** Cushing syndrome, gestation, osilodrostat

## Abstract

Osilodrostat is a novel treatment for adrenocorticotropin-dependent Cushing syndrome; however, its safety during pregnancy has not been reported. This case involves a patient with Cushing disease who became pregnant while on osilodrostat. She was diagnosed at 31 years of age and underwent pituitary tumor removal. After a relapse at 35 years of age, she was initially treated with metyrapone but switched to osilodrostat and hydrocortisone because of nausea, achieving reasonable cortisol control. At 37 years of age, she unknowingly became pregnant despite irregular periods, and the pregnancy was detected at 16 weeks because of ongoing nausea. Osilodrostat was stopped, and she was started on pasireotide and metyrapone. The pregnancy proceeded normally despite elevated urinary free cortisol levels, although she contracted COVID-19 at 25 weeks. At 26 weeks and 1 day, preterm rupture of membranes and breech presentation led to an emergency cesarean section. The newborn had no adrenal insufficiency and developed normally. This case prompts consideration of whether osilodrostat can be used during pregnancy if risks are justified. Pasireotide is rarely used in pregnancy and may have limited effectiveness, but when given, can cause hyperglycemia because of insulin and incretin suppression and should be monitored carefully.

## Introduction

Active Cushing syndrome decreases fertility, which explains its rarity in pregnancy. Fewer than 250 cases have been documented [1]. Whether it is ACTH-dependent or ACTH-independent, this disease poses significant risks to both mother and fetus. Its maternal complications include hypertension, preeclampsia, and diabetes [2], whereas the fetal risks include miscarriage, intrauterine growth restriction, and prematurity [3]. Given its rarity, there is no established standard of care for Cushing disease during pregnancy. Surgery offers a potential cure, but it can cause hypopituitarism and may not be feasible in the absence of a visible tumor [4]. Meanwhile, there are also risks associated with radiotherapy and pharmacological treatments [1, 4]. The use of pasireotide, a somatostatin analog, for the treatment of a GH-secreting pituitary macroadenoma without complications has been reported in only 1 case during pregnancy [5]. To the best of our knowledge, this drug has not been used for Cushing disease before. Osilodrostat, like metyrapone, is a newer steroidogenesis inhibitor that blocks 11β-hydroxylase in the adrenal glands. It is effective for both ACTH-dependent and ACTH-independent Cushing syndrome [6]. However, it is contraindicated in pregnancy because of its proven teratogenic effects in animal studies [7]. As a result, data on its use in human pregnancy are lacking. Understanding the normal physiology of the hypothalamic-pituitary-adrenal (HPA) axis in pregnancy is essential. In normal pregnancy, the maternal levels of corticotropin-releasing hormone, ACTH, and cortisol rise both in the serum and urine because of placental production [8, 9]. Although cortisol levels rise, only about 10% crosses the placenta because of 11β-hydroxysteroid dehydrogenase activity [10]. Fetal cortisol production remains minimal until late gestation, as 3β-hydroxysteroid dehydrogenase activity stays low until then [10]. Thus, most fetal cortisol originates from maternal sources [11]. In late pregnancy, fetal adrenal 3β-hydroxysteroid dehydrogenase activity increases, thereby enhancing fetal cortisol synthesis and promoting maturation of the HPA axis [10]. This case report discusses a female patient with recurrent Cushing disease who conceived while taking osilodrostat, which she took until early pregnancy; she was later treated successfully with pasireotide and metyrapone.

## Case Presentation

**Figure 1.**
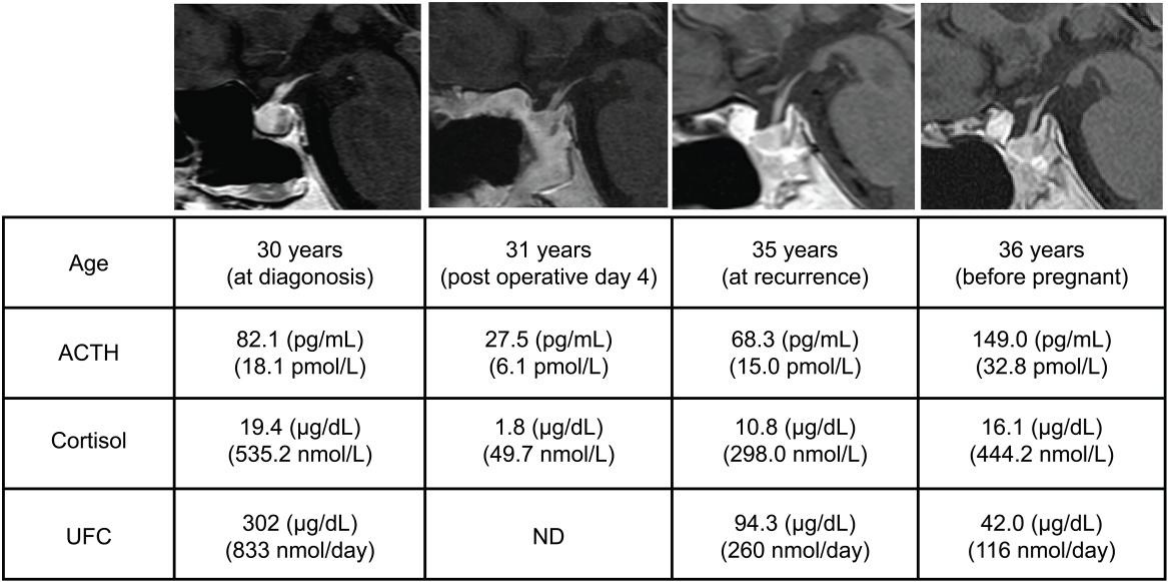
Changes in urinary free cortisol (UFC) and pituitary magnetic resonance imaging (MRI) findings over time. The MRI scans at diagnosis, after surgery, at recurrence, and before pregnancy are shown alongside ACTH, cortisol, and UFC levels. The blood tests indicated recurrence, but no tumor was seen on MRI. Cortisol levels improved after osilodrostat treatment.

A 30-year-old woman developed moon facies, central obesity, muscle weakness, and amenorrhea. Elevated levels of ACTH and cortisol, along with a roughly 6-mm pituitary adenoma, confirmed a diagnosis of Cushing disease. At 31 years of age, she successfully underwent transsphenoidal surgery, but 4 years later, biochemical relapse occurred with no identifiable residual tumor on imaging (Fig. 1). The patient was initially treated with metyrapone, but because of nausea, this was switched to osilodrostat. A block-and-replace approach was taken with osilodrostat 3 mg/day and hydrocortisone 10 mg/day, after which her cortisol levels normalized, but the menstrual irregularities persisted (Fig. 1).

## Diagnostic Assessment

At 38 years of age, the patient presented with nausea. The patient was followed up with an upper gastrointestinal endoscopy revealing no abnormalities. After a prolonged period of nausea, a pregnancy test revealed that she was 16 weeks pregnant.

## Treatment

**Figure 2.**
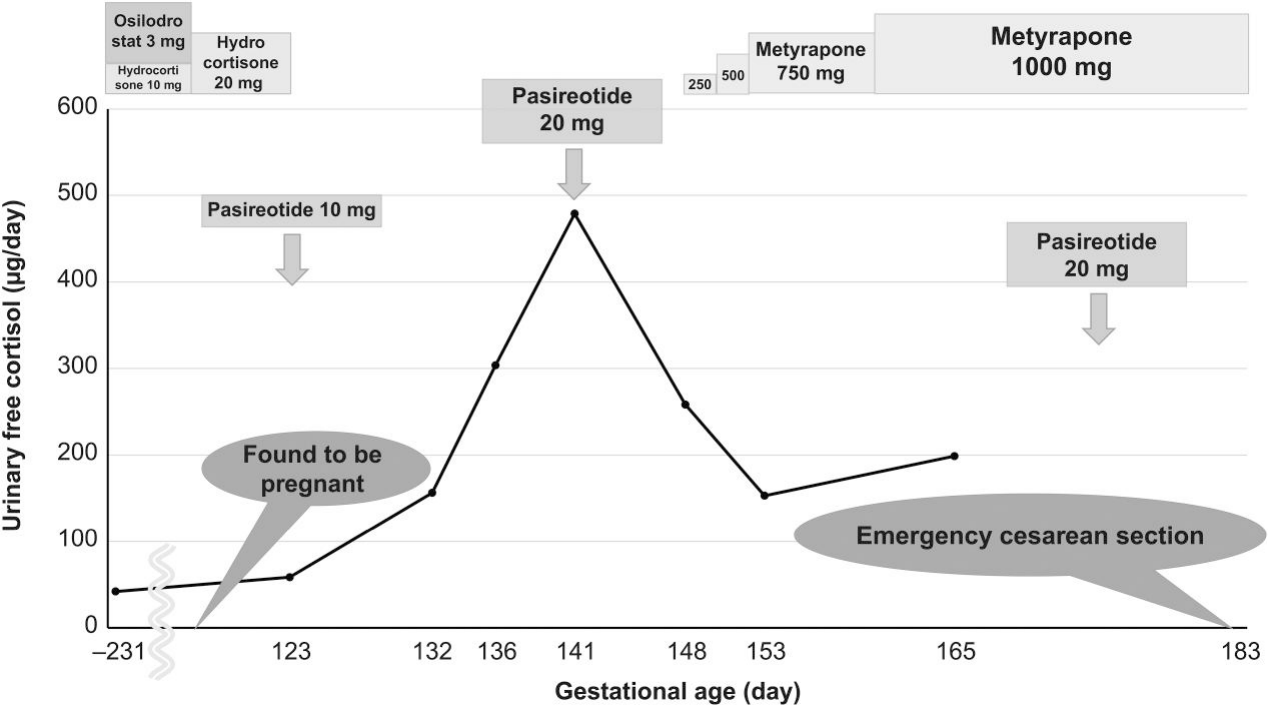
Urinary free cortisol (UFC) levels and medications during pregnancy. The UFC levels during pregnancy are shown. The UFC levels increased after stopping osilodrostat, and these remained high even after starting pasireotide. Adding metyrapone led to a decrease in the UFC.

At this point, she had been on osilodrostat, which was immediately stopped and replaced with pasireotide 10 mg every 4 weeks because of pregnancy. Later, 24-hour urinary free cortisol (UFC) levels increased, leading to an early increase in pasireotide dose to 20 mg after 3 weeks before the recommended 4-week period elapsed; the same dose was administered every 4 weeks thereafter. And the same time, the initiation of up to 1000 mg metyrapone daily (Fig. 2). The patient also had hyperglycemia, which prompted insulin initiation, and subcutaneous heparin was also added because of the risk of thrombosis. At 25 weeks of pregnancy, she developed pharyngeal pain and a cough, which quickly resolved. At 26 weeks and 1 day, she experienced preterm premature rupture of membranes with the fetus in breech position, necessitating an emergency cesarean section. During this time, she tested positive for severe acute respiratory syndrome coronavirus 2 via polymerase chain reaction; however, she remained asymptomatic. Hydrocortisone was given before delivery as a steroid cover. Postpartum, osilodrostat was resumed, and pasireotide/metyrapone was discontinued. Two months after delivery, her disease remained stable, with UFC at 62.0 μg/day (171 nmol/day), within the normal reference range of 26.0 to 187.0 μg/day (72-516 nmol/day).

## Outcome and Follow-up

A live baby girl was born with extremely low birth weight, weighing 871 g. She was admitted to the neonatal intensive care unit with Apgar scores of 2 and 10 at 1 and 5 minutes, respectively, and was temporarily placed on a ventilator because of respiratory distress syndrome. During her stay, no signs of adrenal insufficiency appeared, and blood samples taken at noon showed ACTH levels of 23.3 pg/mL (5.1 pmol/L) and cortisol levels of 2.7 µg/dL (74.5 nmol/L). The normal reference ranges in adults are 7.2 to 63.3 pg/mL (1.6-13.9 pmol/L) for ACTH and 4.5 to 21.1 µg/dL (124.2-582.1 pmol/L) for cortisol. She was discharged at 40 weeks’ corrected gestational age, with subsequent normal growth and development.

## Discussion

It remains challenging to manage Cushing disease during pregnancy because of limited treatment options and fetal safety concerns. An important aspect of managing hypercortisolemia in pregnancy is understanding the physiological regulation of the maternal-fetal HPA axis. In infants with very low birth weight, cortisol levels measured within an hour after birth typically range from 3.6 to 10.8 µg/dL (99-298 nmol/L) [12]. Although the neonate in this case had lower cortisol levels (2.7 µg/dL, 74.5 nmol/L), the blood sample was taken around noon, a time when levels are usually lower. Nevertheless, no signs of adrenal insufficiency were observed. Because newborns develop a stable cortisol rhythm within the first month [13], these findings suggest adequate adrenal function. Better obstetric outcomes can be expected when maternal hypercortisolism is successfully managed, such as reduced rates of prematurity and low birth weight [14]. A previous case report noted successful delivery after treatment with metyrapone, targeting UFC levels below 150 µg/day (414 nmol/day) [15]. Metyrapone was necessary in this patient because the cortisol levels were rising despite pasireotide monotherapy. This was gradually titrated to control UFC levels, which achieved some success. We introduced pasireotide during pregnancy based on previous reports of its use in acromegaly without adverse fetal outcomes [5]. However, pasireotide carries significant risk of hyperglycemia because of its inhibitory effects on insulin and incretin secretion [16]; this was seen in our patient, who required insulin therapy. Although rarely used in pregnancy—with only 1 reported case to our knowledge—it may be considered a viable option if other treatments are unsuccessful or unsuitable. Osilodrostat is contraindicated during pregnancy because it has shown teratogenic effects in animal studies, leading to limited human data [6]. In this case, the patient was unknowingly exposed during early pregnancy. However, no fetal malformations were observed, and this could be attributed to the underdeveloped fetal adrenal cortex during early gestation, which mainly relies on maternal hormone supply [10]. Osilodrostat was resumed after delivery, achieving effective disease control and clinical stability. It is also essential to consider that the preterm birth in this case may have resulted from suboptimal cortisol control, maternal COVID-19 infection, and the use of osilodrostat and pasireotide—drugs with minimal clinical data for use during pregnancy. These factors cannot be excluded entirely. However, based on our expertise, the contraindication of osilodrostat in pregnancy may warrant reevaluation.

## Learning Points

- Osilodrostat should not be used during pregnancy. Although preterm birth in this case may have resulted from various factors—including limited clinical data on osilodrostat and pasireotide—that the neonate showed no congenital abnormalities or adrenal problems indicates that the current caution against using osilodrostat in pregnancy might need to be reconsidered.
- In early pregnancy, the fetal adrenal glands are immature and dependent on maternal hormones, so the effects of drugs that inhibit adrenal steroid synthesis may be relatively minor.
- Pasireotide is rarely used during pregnancy. If administered, close monitoring is necessary, as insulin and incretin suppression may induce hyperglycemia.

## Data Availability

The original data generated and analyzed during this study are included in this published article.
